# HLA class II genotyping of admixed Brazilian patients with type 1 diabetes according to self-reported color/race in a nationwide study

**DOI:** 10.1038/s41598-020-63322-y

**Published:** 2020-04-20

**Authors:** Deborah Conte Santos, Luís Cristóvão Porto, Romulo Vianna Oliveira, Danielle Secco, Leonardo Hanhoerderster, Marcela Haas Pizarro, Bianca S. V. Barros, Laura G. N. Mello, Luiza Harcar Muniz, Dayse A. Silva, Marília Brito Gomes

**Affiliations:** 1 Department of Internal Medicine, Diabetes Unit, Rio de Janeiro State University (UERJ), Rio de Janeiro, Rio de Janeiro Brazil; 2 Histocompatibility and Cryopreservation Laboratory (HLA), Rio de Janeiro State University (UERJ), Rio de Janeiro, Rio de Janeiro Brazil; 3 DNA Diagnostic Laboratory (LDD), Rio de Janeiro State University (UERJ), Rio de Janeiro, Rio de Janeiro Brazil

**Keywords:** Clinical genetics, Endocrine system and metabolic diseases

## Abstract

The HLA region is responsible for almost 50% of the genetic risk of type 1 diabetes (T1D). However, haplotypes and their effects on risk or protection vary among different ethnic groups, mainly in an admixed population. We aimed to evaluate the HLA class II genetic profile of Brazilian individuals with T1D and its relationship with self-reported color/race. This was a nationwide multicenter study conducted in 10 Brazilian cities. We included 1,019 T1D individuals and 5,116 controls matched for the region of birth and self-reported color/race. Control participants belonged to the bone marrow transplant donor registry of Brazil (REDOME). HLA-class II alleles (DRB1, DQA1, and DQB1) were genotyped using the SSO and NGS methods. The most frequent risk and protection haplotypes were HLA~DRB1*03:01~DQA1*05:01 g~DQB1*02:01 (OR 5.8, p < 0.00001) and HLA~DRB1*07:01~DQA1*02:01~DQB1*02:02 (OR 0.54, p < 0.0001), respectively, regardless of self-reported color/race. Haplotypes HLA~DRB1*03:01~DQA1*05:01 g~DQB1*02:01 and HLA~DRB1*04:02~DQA1*03:01 g~DQB1*03:02 were more prevalent in the self-reported White group than in the Black group (p = 0.04 and p = 0.02, respectively). The frequency of haplotype HLA*~DR*B1*09:01~DQA1*03:01 g~DQB1*02:02 was higher in individuals self-reported as Black than White (p = <0.00001). No difference between the Brazilian geographical regions was found. Individuals with T1D presented differences in frequencies of haplotypes within self-reported color/race, but the more prevalent haplotypes, regardless of self-reported color/race, were the ones described previously in Europeans. We hypothesize that, in the T1D population of Brazil, although highly admixed, the disease risk alleles come mostly from Europeans as a result of centuries of colonization and migration.

## Introduction

Type 1 diabetes (T1D) is a chronic polygenic disease that arises from the combination of multiple genetic and environmental factors^1^. The HLA region on chromosome 6p21 is known to be responsible for almost 50% of the genetic risk, and it has been associated with diabetes since the 1970s^2^. Even though HLA Class I genes and non-HLA genes also contribute to T1D risk, Class II alleles such as DR and DQ demonstrate the strongest associations with the disease^1,3^.

Susceptibility to T1D is mainly associated with haplotype *DR*B1*04~DQA1*03:01~DQB1*03:02, followed by DRB1*03:01*~DQ*A1*05:01*~DQ*B1*02:01^3^. More than 90% of patients carry one of those two haplotypes, and 30% carry both of them, while the prevalence in the general population is 2%^4–6^, including previous data from regional populations and familial studies from Brazil^7–9^.

In recent years, several risk scores for the diagnosis and risk assessment of T1D have been proposed, using growing and extensive knowledge about T1D genetics^1^. Most of them are based on the presence of high-risk HLA alleles described in previous studies^10–12^.

The highest prevalence of T1D is observed in the European population^13^, and most of the studies are concentrated on homogeneous populations^6,14–16^. However, previous data have shown that the frequency of HLA haplotypes, as well as their effects on T1D risk or protection, could vary among populations^17^. With the advancement of genetic risk scores for the diagnosis and prediction of T1D, it is critical to account for ethnic differences in the genetics of T1D that may impact clinical outcomes such as chronic complications. Haplotypes that denote risk for one population might have a phenotype of protection on another. For instance, the haplotype DRB1*07:01~DQA1*03:01~DQB1*02:02 appears to be protective for the European population and denotes susceptibility for African Americans^18^.

Brazil has a large multiethnic population as a result of centuries of miscegenation since Portuguese colonization in 1500. The Brazilian population is formed by basically three principal ancestry roots: European (EUR), sub-Saharan African (AFR), and Native American (NAM). The country was originally populated by NAM. With the colonization, and later de slavery traffic, the EUR (particularly Portuguese) and the AFR ancestries started the miscegenation of the population, spreading gradually to the internal part of the country, explaining the substantial Brazilian genetic variability^19–21^.

There is a scarcity of data on the genetics of the T1D population in Brazil, characterized as highly admixed. In this study, the primary objective was to evaluate the HLA class II genetic profile of Brazilian individuals with T1D and its relationship with self-reported color/race (CRsr) in comparison to a sample of individuals without T1D that belonged to the bone marrow transplant donor’s registry of Brazil (REDOME), matched by region of birth and self-reported color/race. Second, we aimed to analyze regional geographic differences in HLA class II risk distribution of individuals with T1D in Brazil, a country with continental proportions.

## Research design and methods

### Study design and population

This analysis derives from a nationwide multicenter cross-sectional study conducted between August 2011 and August 2014 in 14 public clinics located in 10 Brazilian cities. The methods have been described previously^22^. Briefly, subjects received health care from the National Brazilian Health Care System (SUS) and were included in the study if they had been diagnosed by the presence of typical clinical presentation of T1D, including variable degrees of hyperglycemia, weight loss, polyuria, polydipsia, polyphagia and the need for continuous insulin use since the diagnosis with at least six months of follow-up evaluations in each center. From the initial cohort of 1,760, we randomly selected 1,019 individuals by region of birth and CRsr. The comparison between the selected group and the initial group showed no differences regarding principal clinical and demographic variables (data not shown). The institutional ethics committee of Pedro Ernesto University Hospital (State University of Rio de Janeiro) and each center’s local ethics committee approved the study. All participants or their representatives signed written informed consent for the study. A standardized questionnaire was also used during a clinical visit to evaluate clinical and demographic data such as gender, current age, birthplace, self-reported color/race, age at diagnosis and duration of diabetes.

We also included information on HLA typing, region of birth and CRsr from 5,116 REDOME entries matched for the region of birth and CRsr at a 5:1 ratio. Inclusion criteria as a donor at REDOME are 18 to 55 years of age, good health status, and no infection, hematological or immunological disease. Individuals who had a diagnosis of cancer or diabetes with the use of insulin or other injectable medication are also excluded from REDOME^23^. We provide a supplemental figure with a chart flow of the selection process of patients with and without T1D (Supplemental Fig. S1). Each center’s local ethics committee approved the study. All participants or their representatives signed written informed consent for the study. A standardized questionnaire was also used during a clinical visit to evaluate clinical and demographic data such as gender, current age, birthplace, self-reported color/race, age at diagnosis, and duration of diabetes.

### Self-reported color/race

Participants categorized themselves into one of the five CRsr groups used for the Brazilian population censuses: White (“branca”), Brown (“parda”), Black (“preta”), Asian (yellow “amarela”), and Indigenous (“indígena”) as recommended by IBGE^24^.

### DNA extraction

Genomic DNA was extracted from peripheral blood with the commercial SP QIA Symphony Kit by automation with QIA Symphony equipment, following the manufacturer’s instructions (Qiagen, USA).

### HLA genotyping

HLA-class II alleles (*DRB1*, *DQA1*, and *DQB1*) from 1,019 individuals with T1D were genotyped. Genotyping was performed using PCR-RSSO (LabType SSO2B1 High resolution, One Lambda Inc., West Hills, USA) in 543 (53.3%) participants with T1D and 476 (46.7%) had their DNA typed by next-generation sequencing (NGS). Of those, 352 were amplified at loci *HLA-DRB1* and *HLA-DQB1* by long-range PCR using primers from the NGSgo.v2 (GenDx, Utrecht, the Netherlands) Library Preparation Kit and 124 with Holotype HLA Assay (Omixon Inc., Budapest, Hungary) for *HLA-DR*B1, *HLA-DQ*B1 and *HLA-DQ*A1, according to the manufacturer’s instructions. These primers cover exons 2, 3, and 4. *HLA-DQA1* allele was imputed in 31.5% of the samples from the group of T1D individuals (n = 321) using the linkage disequilibrium criteria, based on the results found by NGS.

The HLA genotyping results of the group of participants without T1D were obtained at high resolution in DRB1 and DQB1 loci in 2,201 REDOME entries. The class II alleles assigned in any loci with NMDP codes were defined based on Common and Well Documented, version 2.0 (n = 2,915). *HLA-DQ*A1 alleles were typed in all control samples with PCR-RSSO (LabType SSO2B1 High resolution, One Lambda Inc., West Hills, USA).

Ambiguous HLA class II alleles within the P or G group were designated by a lower case ‘g’ (DRB1*12:01 *g* = 12:01/12:10; DQA1*01:01 *g* = 01:01/01:04/01:05; DQA1*03:01 *g* = 03:01/03:02/03:03; DQA1*05:01 *g* = 05:05/05:09; DQB1*03:01 *g* = 03:01/03:09/03:19). After validating the HLA dataset via an EM algorithm for resolving allelic ambiguities and determining both allele and extended haplotype frequencies despite some missing loci data, this imputation was manually performed according to the haplotype results from Arlequin output data for both groups (individuals with and without T1D), according to race and region.

Three-locus haplotype frequencies (*DRB1~DQA1~DQB1*) were estimated for each of the races and regions for both groups (individuals with and without T1D), resolving phase and allelic ambiguity using the expectation-maximization (EM) algorithm^25,26^. Deviations from Hardy-Weinberg equilibrium (HWE) were assessed at the allele-family level (first nomenclature field) using a modified version of the Guo and Thompson algorithm^27^ as implemented in Arlequin software v.3.5^28^.

The most frequent haplotypes associated with risk for T1D (OR > 3.0) were compared among Brazilian regions.

### Statistical analysis

Categorical variables such as self-reported color/race, geographical region of birth and gender were presented as frequencies (percentages). All normally distributed continuous variables, such as age, duration of diabetes, and HbA1c values, were given as the mean ± standard deviation (SD). We used chi-square and Fisher’s tests to compare categorical data; Student’s t-test and analysis of variance (ANOVA) were used for comparisons between groups with numeric variables when indicated. Samples were divided into two groups (individuals with and without T1D) for population comparison testing. Arlequin software was used to calculate FST genetic distance, and the exact test for population differentiation test results was performed via allele frequency extrapolations^28^. Tests were then repeated after dividing the two populations into smaller groups according to self-declared ethnicity (White, Black, and Brown, when n > 30) and region to detect potential ancestry or regional related biases.

Bonferroni correction was applied for multiple tests. We used the Statistical Program for Social Sciences version 17.0 (SPSS, Inc., Chicago, Illinois). A two-sided p-value of less than 0.05 was considered significant. Haplotype frequencies between cases and controls were compared using a Pearson chi-square test. Odds ratios (ORs) and 95% CIs were calculated.

## Results

### Population characteristics

Population characteristics are shown in Table 1. Half of the participants declared themselves as White. Individuals with T1D were older than healthy participants (p = 0.02). The group of individuals without diabetes had more male individuals than the group of individuals with T1D (p < 0.001).

**Table 1:** Population characteristics.

	T1D Individuals (1019)	Individuals without T1D (5116)	p-value
**Self-reported color/race**			0.37
White	520 (51.0%)	2625 (51.3%)	
Brown	382 (37.5%)	1948 (38.1%)	
Black	94 (9.2%)	464 (9.1%)	
Asian	11 (1.1%)	48 (0.9%)	
Indigenous	12 (1.2%)	31 (0.6%)	
**Region**			0.99
Midwest	81 (7.9%)	397 (7.8%)	
Northeast	318 (31.2%)	1587 (31.0%)	
North	44 (4.3%)	223 (4.4%)	
Southeast	429 (42.1%)	2173 (42.5%)	
South	147 (14.4%)	736 (14.4%)	
**Gender**			<0.001
Female	563 (55.3%)	2304 (45%)	
Male	456 (44.7%)	2812 (55%)	
**Age**	30.2 ± 11.8	29.4 ± 8.8	0.02
**Diabetes duration**	15.3 ± 8.9		
**HbA1c %**	8.9 ± 2.4		
**HbA1c mmol**	74.7 ± 25.0		

Data are represented as number (%) or mean ± SD (standard deviation). T1D = type 1 diabetes.

### Overview of the risk and protective alleles and/or haplotypes of the HLA system in individuals with and without T1D

The frequencies of the HLA-DRB1, HLA-DQB1, and HLA-DQA1 alleles are shown in Tables 2–4, respectively. The *HLA-DRB1* alleles associated with the risk of T1D were DRB1*03 (OR 4.03, CI 3.60–4.51, p < 0.0001) and DRB1*04 (OR 2.98, CI 2.66–3.33, p < 0.0001) and DRB1*09 (OR 2.43, CI 1.84–3.19, p < 0.001). The most frequent protective *HLA-DRB1* alleles were DRB1*13 (OR 0.56, CI 0.47–0.66, p < 0.001), DRB1*07 (OR 0.49, CI 0.41–0.59, p < 0.0001) and DRB1*11 (OR 0.26, CI 0.20–0.33, p < 0.p < 0.001). Only the HLA-DQA1*03 allele conferred risk (OR 3.24, p < 0.001). The two *HLA-DQB1* alleles that were associated with the risk of T1D were HLA-DQB1*02 (OR 2.93, CI 2.64–3.24, p < 0.0001) and DQB1*03 (OR 1.38, CI 1.25–1.53, p < 0.001). HLA-DQA1*03 (OR 3.24, CI 2.91–3.61, p < 0.0001) and DQA1*05 (OR 2.28, CI 2.05–2.53, p < 0.0001) alleles conferred risk in our population of T1D.

**Table 2:** *HLA-DRB1* alleles distribution in individuals with type 1 diabetes and without T1D.

DRB1*	Individuals without T1D N (%)	Individuals with T1D N (%)	OR	CI	P-value
*01*	1,012 (9.89%)	151 (7.41%)	0.73	0.61–0.87	0.000
*03*	1,014 (9.91%)	626 (30.72%)	4.03	3.60–4.51	0.000
*04*	1,283 (12.54%)	610 (29.93%)	2.98	2.66–3.33	0.000
*07*	1,321 (12.91%)	139 (6.82%)	0.49	0.41–0.59	0.000
*08*	644 (6.29%)	67 (3.29%)	0.51	0.39–0.65	0.000
*09*	163 (1.59%)	77 (3.78%)	2.43	1.84–3.19	0.000
*10*	202 (1.97%)	24 (1.18%)	0.59	0.39–0.91	0.015
*11*	1,211 (11.84%)	69 (3.39%)	0.26	0.20–0.33	0.000
*12*	162 (1.58%)	19 (0.93%)	0.58	0.36–0.94	0.026
*13*	1,397 (13.65%)	165 (8.10%)	0.56	0.47–0.66	0.000
*14*	415 (4.06%)	15 (0.74%)	0.17	0.10–0.29	0.000
*15*	1,027 (10.04%)	44 (2.16%)	0.2	0.15–0.27	0.000
*16*	381 (3.72%)	32 (1.57%)	0.41	0.28–0.59	0.000

T1D = type 1 diabetes mellitus; n = number of individuals; OR = odds ratio; CI = confidence interval; DRB1*01 included 01:01, 01:02, 01:03 and 01:28; DRB1*03 included 03:01, 03:02, 03:05, 03:07, 03:11, 03:12, 03:15, 03:37, 03:52 and 03:61; DRB1*04 included 04:01, 04:02, 04:03, 04:04, 04:05, 04:06, 04:07, 04:08, 04:09, 04:10, 04:11, 04:14, 04:29 and 04:50; DRB1*07 included 07:01, 07:11 and 07:15; DRB1*08 included 08:01, 08:02, 08:03, 08:04, 08:06, 08:07 and 08:10; DRB1*09 included 09:01 and 09:10; DRB1*10 included 10:01 and 10:03; DRB1*11 included 11:01, 11:02, 11:03, 11:04, 11:06, 11:11, 11:13, 11:18, 11:19, 11:34 and 11:37; DRB1*12 included 12:01, 12:01 G, 12:02, 12:05 and 12:38; DRB1*13 included 13:01, 13:02, 13:03, 13:04, 13:05, 13:11, 13:15, 13:23, 13:31, 13:40, 13:42, 13:49 and 13:56; DRB1*14 included 14:01, 14:02, 14:03, 14:04, 14:06, 14:07, 14:13, 14:17, 14:21, 14:54 and 14:81; DRB1*15 included 15:01, 15:02, 15:03, 15:18 and 15:20; DRB1*16 included 16:01 and 16:02.

**Table 3:** *HLA-DQB1* alleles distribution in individuals with type 1 diabetes and without T1D.

DQB1*	T1D Individuals N (%)	Individuals with T1D N (%)	OR	CI	P value
*02*	1,969 (19.24%)	837 (41.07%)	2.93	2.64–3.24	0.000
*03*	2,873 (28.08%)	715 (35.08%)	1.38	1.25–1.53	0.000
*04*	892 (8.72%)	64 (3.14%)	0.34	0.26–0.44	0.000
*05*	1,901 (18.58%)	243 (11.92%)	0.59	0.51–0.68	0.000
*06*	2,597 (25.38%)	179 (8.78%)	0.28	0.24–0.33	0.000

T1D = type 1 diabetes mellitus; n = number of individuals; OR = odds ratio; CI = confidence interval, DQB1*02 included 02:01, 02:02, 02:03; DQB1*03 included 03:01 G, 03:02, 03:03, 03:04, 03:05, 03:14, 03:34, 03:40/03:110/03:141/03:155, 03:10/03:139/03:186 and 03:41; DQB1*04included 04:01, 04:02 and 04:04; DQB1*05 included 05:01, 05:02, 05:03, 05:04, 05:05, 05:07, 05:11 and 05:47/05:165; DQB1*06 included 06:01, 06:02, 06:03, 06:04, 06:05, 06:08, 06:09, 06:10, 06:11, 06:19, 06:26 N, 06:27, 06:33, 06:38, 06:49 and 06:72.

**Table 4:** HLA-DQA1 alleles distribution in patients with type 1 diabetes and without T1D.

DQA1*	Individuals without T1D N (%)	Individuals with T1D N (%)	OR	CI	P-value
*01*	4,491 (43.89%)	415 (20.36%)	0.33	0.29–0.37	0.000
*02:01*	1,351 (13.20%)	134 (6.58%)	0.46	0.38–0.56	0.000
*03:01* *g*	1,417 (13.85%)	698 (34.25%)	3.24	2.91–3.61	0.000
*04*	873 (8.53%)	72 (3.53%)	0.39	0.31–0.50	0.000
*05*	1,974 (19.29%)	718 (35.23%)	2.28	2.05–2.53	0.000
*06:01*	126 (1.23%)	1 (0.05%)	0.04	0.01–0.28	0.000

T1D = type 1 diabetes mellitus; n = number of individuals; OR = odds ratio; CI = confidence interval; DQA1*01 included 01:01 g, 01:02, 01:03, 01:07 and 01:13; DQA1*04 included 04:01, 04:02, 04:03 and 04:04; DQA1*05 included 05:01 g, 05:02, 05:03, 05:04, 05:08 and 05:10.

Table 5 shows the frequencies of DRB1/DRB1 genotypes in both groups. The HLA-DRB1*03/DRB1*04 genotype presented the highest risk (OR 12.1, CI 9.64–15.20, p < 0.0001) in 23.6% of the T1D participants, followed by DRB1*03/DRB1*03 (OR 10.6, CI 7.54–14.92, p < 0.0001) in 9.8% and DRB1*03/DRB1*09 (9.01, CI 4.85–16.71, p < 0.0001) in 2.7%.

**Table 5:** *HLA-DRB1/DRB1* genotypes distribution in individuals with type 1 diabetes and without T1D.

Genotypes	T1D Individuals with T1D N (%)	Individuals without T1D N (%)	OR	CI	p value
DRB1*01/DRB1*XX	95 (9.32%)	1862 (36.40%)	0.18	0.14–0.22	0.00000
DRB1*03/DRB1*01	40 (3.93%)	99 (1.94%)	2.07	1.42–3.01	0.00010
DRB1*03/DRB1*03	100 (9.81%)	52 (1.02%)	10.6	7.52–14.92	0.00000
DRB1*03/DRB1*04	240 (23.55%)	127 (2.48%)	12.1	9.64–15.20	0.00000
DRB1*03/DRB1*09	28 (2.75%)	16 (0.31%)	9.01	4.85–16.71	0.00000
DRB1*03/DRB1*XX	117 (11.48%)	668 (13.06%)	0.86	0.70–1.07	0.16924
DRB1*04/DRB1*01	47 (4.61%)	128 (2.50%)	1.88	1.34–2.65	0.00022
DRB1*04/DRB1*04	60 (5.89%)	82 (1.60%)	3.84	2.73–5.40	0.00000
DRB1*04/DRB1*09	13 (1.28%)	21 (0.41%)	3.13	1.56–6.28	0.00068
DRB1*04/DRB1*XX	191 (18.74%)	843 (16.48%)	1.17	0.98–1.39	0.07762
DRB1*09/DRB1*09	2 (0.20%)	0 (0.00%)			
DRB1*09/DRB1*XX	25 (2.45%)	121 (2.37%)	1.04	0.67–1.61	0.86596
DRB1*XX/DRB1*XX	61 (5.99%)	1109 (21.68%)	0.23	0.18–0.30	0.00000

T1D = type 1 diabetes mellitus; n = number of individuals; OR = odds ratio; CI = confidence interval; p required for significance after Bonferroni correction 0.004. DRB1*XX = any haplotype other than DRB1*03, DRB1*04 or DRB1*09.

Frequencies of the full haplotype (*DRB1~DQA1~DQB1*) for both groups are shown in Table 6. Considering a p-value of 0.0007 after Bonferroni correction, 21 of the 66 haplotypes showed a statistically significant association with T1D (positive or negative). The most frequent risk haplotypes found in our population were DRB1*03:01~DQA1*05:01 g~DQB1*02:01 (OR 5.8, CI 5.13–6.57, p < 0.00001), DRB1*04:05~DQA1*03:01 g~DQB1*03:02 (OR 5.34, CI 4.37–6.51, p < 0.00001), and DRB1*04:02~DQA1*03:01 g~DQB1*03:02 (OR 3.43, CI 2.74–4.31, p < 0.00001). The most prevalent protection haplotypes were DRB1*07:01~DQA1*02:01~DQB1*02:02 (OR 0.54, CI 0.44–0.65, p < 0.0001), DRB1*13:01~DQA1*01:03~DQB1*06:03 (OR 0.30, CI 0.22–0.42, p < 0.00001) and DRB1*01:02~DQA1*01:01 g~DQB1*05:01 (OR 0.45, CI 0.34–0.60, p < 0.00001). Table 6 shows haplotypes that were seen at least 18 times total in the T1D participants and the healthy control group. Other less frequent haplotypes were grouped as others.

**Table 6:** Distribution of the *HLA-DRB1~DQA1~DQB1* haplotypes in individuals with type 1 diabetes mellitus and without T1D.

Haplotypes (*DRB1~DQA1~DQB1*)	T1D Individuals N (%)	Individuals without T1D N(%)	OR	CI	p value
*01:01~01:01* *g~05:01*	75 (3.68%)	315 (3.08%)	1.2	0.93–1.55	0.16472
*01:02~01:01* *g~05:01*	54 (2.65%)	578 (5.65%)	0.45	0.34–0.60	**0.00000**
*01:03~01:01* *g~05:01*	4 (0.20%)	67 (0.66%)	0.3	0.11–0.82	0.00970
*03:01~05:01* *g~02:01*	590 (28.95%)	671 (6.56%)	5.8	5.13–6.57	**0.00000**
*03:02~04:01~04:02*	10 (0.49%)	306 (2.99%)	0.16	0.08–0.30	**0.00000**
*04:01~03:01* *g~03:01* *g*	11 (0.54%)	74 (0.72%)	0.74	0.39–1.41	0.45982
*04:01~03:01* *g~03:02*	102 (4.99%)	81 (0.79%)	6.6	4.91–8.87	**0.00000**
*04:02~03:01* *g~03:02*	130 (6.39%)	199 (1.94%)	3.43	2.74–4.31	**0.00000**
*04:03~03:01* *g~03:02*	7 (0.34%)	33 (0.32%)	1.06	0.47–2.41	0.83218
*04:04~03:01* *g~03:02*	69 (3.39%)	100 (0.98%)	3.55	2.60–4.84	**0.00000**
*04:04~03:01* *g~04:02*	1 (0.05%)	19 (0.18%)	0.26	0.03–1.97	0.23192
*04:05~03:01* *g~02:02*	11 (0.52%)	17 (0.17%)	3.26	1.52–6.98	0.00352
*04:05~03:01* *g~03:02*	205 (10.06%)	210 (2.05%)	5.34	4.37–6.51	**0.00000**
*04:06~03:01* *g~04:02*	2 (0.10%)	36 (0.35%)	0.28	0.07–1.16	0.07690
*04:07~01:03~06:03*	0 (0.00%)	20 (0.20%)			
*04:07~03:01* *g~03:01* *g*	3 (0.15%)	58 (0.57%)	0.26	0.08–0.83	0.00924
*04:07~03:01* *g~03:02*	9 (0.44%)	37 (0.36%)	1.22	0.59–2.54	0.55330
*04:08~03:01* *g~03:01* *g*	5 (0.25%)	20 (0.19%)	1.26	0.47–3.35	0.59360
*04:11~03:01* *g~03:02*	14 (0.69%)	258 (2.52%)	0.27	0.16–0.46	**0.00000**
*04:11~03:01* *g~04:02*	1 (0.05%)	21 (0.20%)	0.24	0.03–1.78	0.15815
*07:01~02:01~02:02*	125 (6.12%)	1112 (10.87%)	0.54	0.44–0.65	**0.00000**
*07:01~02:01~03:03*	4 (0.20%)	166 (1.63%)	0.12	0.04–0.32	**0.00000**
*08:01~04:01~04:02*	30 (1.47%)	171 (1.67%)	0.88	0.59–1.30	0.56217
*08:02~04:01~04:02*	6 (0.29%)	139 (1.36%)	0.21	0.09–0.49	**0.00004**
*08:03~06:01~03:01* *g*	1 (0.05%)	68 (0.66%)	0.07	0.01–0.53	**0.00012**
*08:04~04:01~03:01* *g*	9 (0.44%)	66 (0.64%)	0.68	0.34–1.37	0.35046
*08:04~04:01~04:02*	6 (0.29%)	84 (0.82%)	0.36	0.16–0.82	0.01335
*08:07~04:01~04:02*	4 (0.20%)	52 (0.51%)	0.38	0.14–1.07	0.06935
*09:01~03:01* *g~02:02*	58 (2.86%)	61 (0.60%)	4.88	3.40–7.02	**0.00000**
*09:01~03:01* *g~03:03*	11 (0.53%)	82 (0.80%)	0.67	0.36–1.26	0.26463
*10:01~01:01* *g~05:01*	23 (1.13%)	189 (1.84%)	0.61	0.39–0.94	0.02799
*11:01~01:02~05:02*	1 (0.05%)	51 (0.50%)	0.1	0.01–0.71	0.00210
*11:01~01:02~06:02*	8 (0.39%)	327 (3.19%)	0.12	0.06–0.24	**0.00000**
*11:01~01:02~06:11*	0 (0.00%)	22 (0.22%)			
*11:01~05:01* *g~03:01* *g*	19 (0.93%)	324 (3.16%)	0.29	0.18–0.46	**0.00000**
*11:01~05:10~03:01* *g*	0 (0.00%)	18 (0.18%)			
*11:02~05:01* *g~03:01* *g*	12 (0.59%)	150 (1.46%)	0.4	0.22–0.72	0.00155
*11:02~05:10~03:01* *g*	1 (0.05%)	24 (0.23%)	0.21	0.03–1.54	0.10735
*11:03~05:01* *g~03:01* *g*	5 (0.25%)	50 (0.49%)	0.5	0.20–1.26	0.14891
*11:03~05:10~03:01* *g*	0 (0.00%)	21 (0.20%)			
*11:04~05:01* *g~03:01* *g*	16 (0.79%)	119 (1.16%)	0.67	0.40–1.14	0.16619
*12:01* *g~01:01* *g~05:01*	4 (0.20%)	58 (0.57%)	0.34	0.12–0.95	0.02621
*12:01* *g~05:01* *g~03:01* *g*	12 (0.59%)	35 (0.34%)	1.73	0.89–3.33	0.11396
*12:02~01:02~06:02*	0 (0.00%)	20 (0.20%)			
*12:02~06:01~03:01* *g*	0 (0.00%)	35 (0.34%)			
*13:01~01:02~05:01*	6 (0.29%)	32 (0.31%)	0.94	0.39–2.25	0.99990
*13:01~01:03~06:03*	43 (2.11%)	676 (6.60%)	0.3	0.22–0.42	**0.00000**
*13:01~03:01* *g~03:03*	2 (0.10%)	17 (0.17%)	0.59	0.14–2.56	0.75702
*13:02~01:02~05:01*	9 (0.44%)	36 (0.35%)	1.26	0.60–2.61	0.54621
*13:02~01:02~05:02*	3 (0.15%)	15 (0.15%)	1.00	0.29–3.47	0.99480
*13:02~01:02~06:04*	57 (2.80%)	248 (2.42%)	1.16	0.86–1.55	0.31273
*13:02~01:02~06:09*	15 (0.74%)	122 (1.19%)	0.61	0.36–1.05	0.08778
*13:02~01:03~06:03*	1 (0.05%)	34 (0.34%)	0.15	0.02–1.08	0.02212
*13:03~02:01~02:02*	0 (0.00%)	34 (0.33%)			
*13:03~05:01* *g~03:01* *g*	11 (0.54%)	81 (0.79%)	0.68	0.36–1.23	0.26351
*14:02~05:01* *g~03:01* *g*	1 (0.05%)	227 (2.22%)	0.02	0.003–0.15	**0.00000**
*14:04~01:01* *g~05:03*	4 (0.20%)	18 (0.18%)	1.12	0.38–3.30	0.77609
*14:06~05:01* *g~03:01* *g*	3 (0.15%)	43 (0.42%)	0.35	0.11–1.13	0.07289
*14:54~01:01* *g~05:03*	1 (0.05%)	79 (0.77%)	0.06	0.009–0.45	**0.00001**
*15:01~01:02~05:02*	2 (0.10%)	18 (0.18%)	0.56	0.13–2.40	0.55981
*15:01~01:02~06:02*	16 (0.79%)	414 (4.04%)	0.19	0.11–0.31	**0.00000**
*15:02~01:03~06:01*	2 (0.10%)	45 (0.44%)	0.22	0.05–0.92	0.01755
*15:03~01:02~06:02*	18 (0.88%)	503 (4.92%)	0.17	0.11–0.28	**0.00000**
*16:01~01:02~05:02*	17 (0.83%)	212 (2.07%)	0.4	0.24–0.65	**0.00014**
*16:02~01:02~05:02*	6 (0.29%)	70 (0.68%)	0.43	0.19–0.99	0.04314
*16:02~05:01* *g~03:01* *g*	4 (0.20%)	84 (0.82%)	0.24	0.09–0.65	0.00213
Others	159 (7.80%)	660 (6.45%)	1.23	1.03–1.47	0.02560

T1D = type 1 diabetes mellitus; n = number of individuals; OR = odds ratio; CI = confidence interval; sixty-eight haplotypes with total number in patients plus controls greater than 18 were included (0.3%). P required for statistical significance after Bonferroni correction for multiple tests <0.00074. Rare alleles were included in others.

### HLA class II distribution by self-reported color/race

**Figure 1:**
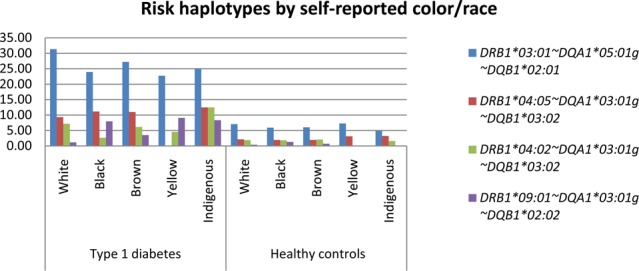
Most relevant risk haplotypes by self-reported color/race.

**Figure 2:**
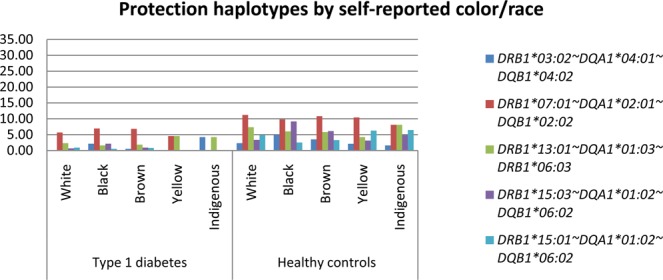
Most relevant protection haplotypes by self-reported color/race.

**Figure 3:**
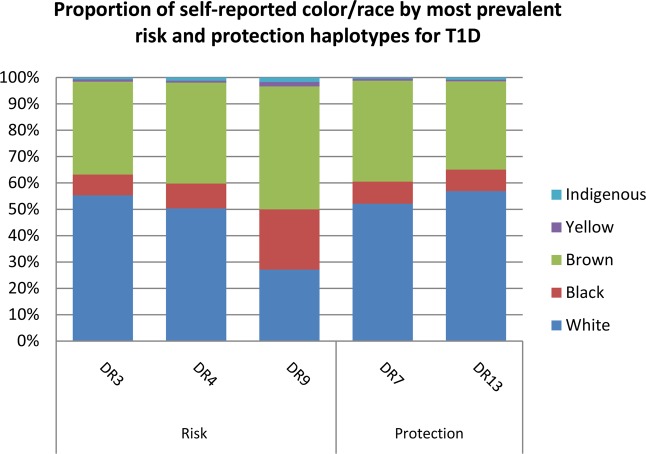
Proportion of self-reported color/race by most prevalent risk and protection haplotypes for T1D. DR3 = DRB1*03:01~DQA1*05:01 g~DQB1*02:01, DR4 = DRB1*04:05~DQA1*03:01 g~DQB1*03:02, DR9 = DRB1*09:01~DQA1*03:01 g~DQB1*02:02, DR7 = DRB2*07:01~DQA1*02:01~DQB1*02:02, DR13 = DRB1*13:01~DQA1*01:03~DQB1*06:03, T1D = Type 1 diabetes.

Figures 1 and 2 present a bar plot with the distribution of the most prevalent risk and protection alleles in the T1D group, respectively, by self-reported color/race. Tables with the haplotype frequencies in both groups stratified by CRsr (White, Black, Brown, Asian and Indigenous) appear in the supplemental material. HLA-DRB1*03:01~DQA1*05:01~DQB1*02:01 was the most frequent risk haplotype in all self-reported color/race groups, and haplotype HLA-DRB1*07:01~DQA1*02:01~DQB1*02:02 was the haplotype with the highest frequency of protection in all groups but did not show statistical significance in the Black, Asian and Indigenous groups. Haplotypes HLA-DRB1*03:01~DQA1*05:01 g~DQB1*02:01 and -DRB1*04:02~DQA1*03:01 g~DQB1*03:02 were significantly more prevalent in the self-declared White group than in the Black group (p = 0.04 and p = 0.02, respectively). Individuals self-reported as Black had a statistically higher prevalence of the haplotype HLA-DRB1* 09:01~DQA1*03:01 g~DQB1*02:02 compared to White and Brown groups (p = <0.00001 and p = 0.008, respectively). This haplotype presented a higher frequency in the Brown group than in the White group (p = 0.001). Figure 3 shows the distribution of the self-reported color/race for the most prevalent risk and protection alleles for all participants. Frequent haplotypes associated with T1D risk grouped by Brazilian regions are shown at Supplemental Table S6. No statistical difference was observed.

## Discussion

In general, our results are in accordance with previous studies in the European population as well as with the last regional studies in Brazil. The most frequent haplotype in all CRsr groups and geographical regions was HLA-DRB1*03:01~DQA1*05:01 g~DQB1*02:01, which is also the most prevalent risk haplotype described in European populations. This demonstrates that although highly admixed, the Brazilian population seems to have greater genetic influence from European populations. The miscegenation process in Brazil is relatively recent, beginning only 500 years ago with the entrance of the Portuguese colonizers (European ancestry). The native Brazilian population was originally formed by indigenous populations, who share some similar HLA alleles and haplotypes with Native Americans^29^. Almost two centuries later, with the beginning of slavery traffic, African ancestry began to contribute to the miscegenation of the Brazilian population. The roots of these three ancestries (European, Native Amerindian, and African) are the basis of our admixed population. Our colonization history described above might explain higher degrees of European ancestry in our population, as demonstrated in previous studies^19^. Although our study design cannot confirm the hypothesis that, in the highly admixed T1D Brazilian population, the disease risk alleles appear to come mostly from Europeans as a result of centuries of colonization and migration, data from two Brazilian previous studies showed that the incidence of T1D was greater in patients self-reported as White^30,31^.

DRB1*03 and DRB1*04 alleles are known to be the most prevalent high-risk alleles between individuals with T1D, with individual allele frequencies varying between 20 and 30%^32^. The highest frequencies are shown in European populations, but they have also been described in African Americans^18^. In Brazil, the frequencies of those alleles are as high as 28%^7^, similar to those found in our sample (28.9%). Up to 63.3% of our type 1 participants carry DRB1*04 and/or DRB1*03, and 39.2% carry both (either in homozygosis or heterozygosis) compared to 5.1% of the control group. It is important to note that the most frequent haplotypes in our analysis were not always the ones with the most significant effect. For instance, although the DRB1*03:01~DQA1*05:01 g~DQB1*02:01 haplotype was the most frequent in individuals with T1D (28.9%), the haplotype with the largest effect was DRB1*04:01~DQB1*03:01 g~DQA1*03:02 (OR 6.6, CI 4.91–8.87, p-value <0.000001).

The commonly described protection alleles are DRB1*03:02, DRB1*07, DRB1*10, DRB1*11, DRB1*13, DRB1*14, *and* DRB1*15. Frequencies of those alleles vary among populations^1^. Haplotype HLA-DRB1*07:01~DQA1*02:01~DQB1*02:02/03:03 was more prevalent in our control group, with a frequency up to 12.5% compared to 6.3% of the T1D group. This haplotype has been described as protective in previous studies in Brazil^32^ as well as in European populations^33^, but it was shown to be associated with risk in the African population^18^. The same situation occurred with DRB1*13. Although the Brazilian population originates mainly from three ancestral roots, with African being one of them, it has lower degrees of sub-Saharan African genomic ancestry than populations presented in the USA, as demonstrated in previous studies from our group^19^. It is important to highlight that up to 51% of our T1D population declared themselves White as opposed to only 9% reported as Black.

Genotype DRB1*03/DRB1*04 presented the highest risk in our study, with an OR of 12.1 (CI 9.64–15.20, p < 0.000001), followed by DRB1*03/DRB1*03 (OR 10.6, CI 7.52–14.92, p < 0.00001). The DRB1*09 allele only presented risk when accompanied by one of the high-risk alleles (DRB1*03 or DRB1*04), and this combination was present in 4% of the individuals with T1D. This result is similar to previous studies in Brazil^7^. A study with the African American population shows DRB1*09 as a risk allele even when not associated with DRB1*03 or DRB1*04^18^. This might be explained by the very low rates of Asian or African ancestry in our population, as discussed above and demonstrated in previous studies^19^. One possible conclusion is that in admixed populations, such as that in Brazil, the disease was brought over by populations of European ancestry, with a stronger presence of DRB1*03 and DRB1*04 among those self-declared as White and the presence of DRB1*09 in those self-reported as Black. Nonetheless, although a frequency variation of the haplotype DRB1*09:01~03:01 *g~02:02* was found among the Brazilian regions within the T1D group (1.4% South vs. 7.8% Northeast), it did not present statistical difference.

It is also important to highlight the rates of homozygosity found in our T1D population, where 9.81% of the individuals with T1D were homozygous for DRB1*03, 5.89% for DRB1*04 and only 0.2% for DRB1*09, similar to a previous study in Brazil^7^. Noble *et al*.’s study in African Americans found similar rates for the DRB1*03 genotype but higher rates for DRB1*09 homozygosity^18^, probably due to the above-cited explanation with a population of higher degrees of African ancestry.

Although we found differences in gender proportions between groups, HLA risk assessment usually does not differ between males and females. One study in children at risk of T1D found an association between gender and HLA risk alleles DRB1*03/DRB1*04 and islet autoimmunity^34^. This is probably not relevant in our population, as we included only individuals with T1D older than 13 years.

In our study, we analyzed only Class II HLA alleles. Although Class I alleles and non-HLA genes also contribute to T1D risk, Class II alleles such as DR and DQ demonstrate the strongest associations with the disease^1^. Recently, several risk scores for diagnosis and risk assessment of T1D have been proposed, and the vast majority of them are based on the presence of high-risk class II HLA alleles^10–12^.

The present study is the first multicenter study in T1D including all five geographical regions of the country with a large multiethnic sample. Additionally, we had a large number of controls matched by region of birth and CRsr, adding strength to our results. Another strength is that we used a uniform, standardized recruitment protocol in all participating centers and the three genotyped loci *HLA*-*DRB1*, *-DQA1*, -and *-DQB1*). REDOME comprises HLA types from all regions and with representative entries of the distinct CRsr, and the allele frequency distributions vary both per region and CRsr^21^. To minimize these differences, a randomized selection included information available in the REDOME database in a pair-matched CSsr and region basis.

Our study has some limitations. First, autoantibodies and C peptide levels were not measured. The diagnosis of diabetes was made based on typical clinical presentation and the need for insulin since diagnosis. Although some individuals with other types of diabetes might have been included, it is important to emphasize that 96.5% of them were diagnosed before 30 years of age, which reinforces the high probability that they most likely have T1D. Second, although T1D participants were from urban areas, patients who receive primary attention care and live in rural areas represent the minority of patients with T1D under treatment in Brazil.

## Conclusion

Regarding the most prevalent risk alleles, such as DRB1*03 and DRB1*04, our findings are in accordance with previous studies both in European and admixed populations. It is important to note that the DRB1*07 allele, which is usually protective only in European populations, was also protective in our population. Additionally, the DRB1*09:01 allele conferred risk only when accompanied by a high-risk allele such as DRB1*03 or DRB1*04. This could be explained by a characteristic of the Brazilian population, in that, although highly admixed, it has a greater contribution of the European ancestry, as demonstrated in previous studies. Therefore, we can conclude that in Brazil, the disease risk allele comes mostly from Europeans. Future studies are needed to better understand the genetics of T1D in admixed populations, especially regarding other genetic loci that might be associated.

### Ethical standard

All procedures performed in the study were in accordance with the ethical standards of the institutional ethics committee of all centers as listed below and with the 1964 Helsinki declaration and its later amendments.

**List of Ethics committee**

Ethics Committee of Pedro Ernesto University Hospital

Ethics Committee of Clementino Fraga Filho University Hospital

Ethics Committee of Clinic’s Hospital of São Paulo’s University Medical School

Ethics Committee of Faculty of Medical Sciences of Campina’s State University

Ethics Committee of Federal University of São Paulo

Ethics committee of the Municipal Health Department of Bauru

Ethics Human Research Committee of João de Barros Barreto University Hospital, Federal University of Pará

Research Ethics Committee of the Diabetes and Endocrinology Center of Bahia

Research Ethics committee of the Federal University of Ceará

Research Ethics committee of the Municipal Health Department of Distrito Federal

Research Ethics Committee of Clinic’s Hospital of Porto Alegre

Ethics Human Research Committee of Federal University of Parana Clinic Hospital

Research Ethics Committee of Walter Cantídio University Hospital

### Informed consent

Informed consent was obtained from all the study participants included in the study, which has done according to the national guidelines of ethical standards and in keeping with the Helsinki Declaration of 2008 (ICH GCP).

## Supplementary information

Supplementary information.
