# Supplementary material for: HLA class II genotyping of admixed Brazilian patients with type 1 diabetes according to self-reported color/race in a nationwide study
Source: Sci Rep. 2020 Apr 20;10:6628. doi: 10.1038/s41598-020-63322-y (PMC7170860; doi:10.1038/s41598-020-63322-y)
Supplement: Supplementary file 1 — Supplementary information. [file 41598_2020_63322_MOESM1_ESM.pdf]

HLA class II genotyping of admixed Brazilian patients with type 1 diabetes according to self-reported color/race in a nationwide study

Deborah Conte Santos[1*], Luís Cristóvão Porto[2], Romulo Vianna Oliveira[2], Danielle Secco[2], Leonardo Hanhoerderster[2], Marcela Haas Pizarro[1], Bianca S. V. Barros[1], Laura G.N. Mello[1], Luiza Harcar Muniz[1], Dayse A. Silva[3], Marília Brito Gomes[1]

[1] Department of Internal Medicine, Diabetes Unit, Rio de Janeiro State University (UERJ), Rio de Janeiro, Rio de Janeiro, Brazil.

[2] Histocompatibility and Cryopreservation Laboratory (HLA), Rio de Janeiro State University (UERJ), Rio de Janeiro, Rio de Janeiro, Brazil.

[3] DNA Diagnostic Laboratory (LDD), Rio de Janeiro State University (UERJ), Rio de Janeiro, Rio de Janeiro, Brazil.

#Brazilian Diabetes Type 1 Study Group (BrazDiab1SG)

*Corresponding author:

Deborah Conte Santos

Department of Internal Medicine, Diabetes Unit, Rio de Janeiro State University (UERJ)

Boulevard 28 de Setembro, 77- 3º andar - Vila Isabel

Rio de Janeiro - RJ, Brazil CEP 20551-030

Phone/Fax number: +552128688224

E-mail: deborahconte@hotmail.com

Figure S1. Flow chart of the selection of cases and controls

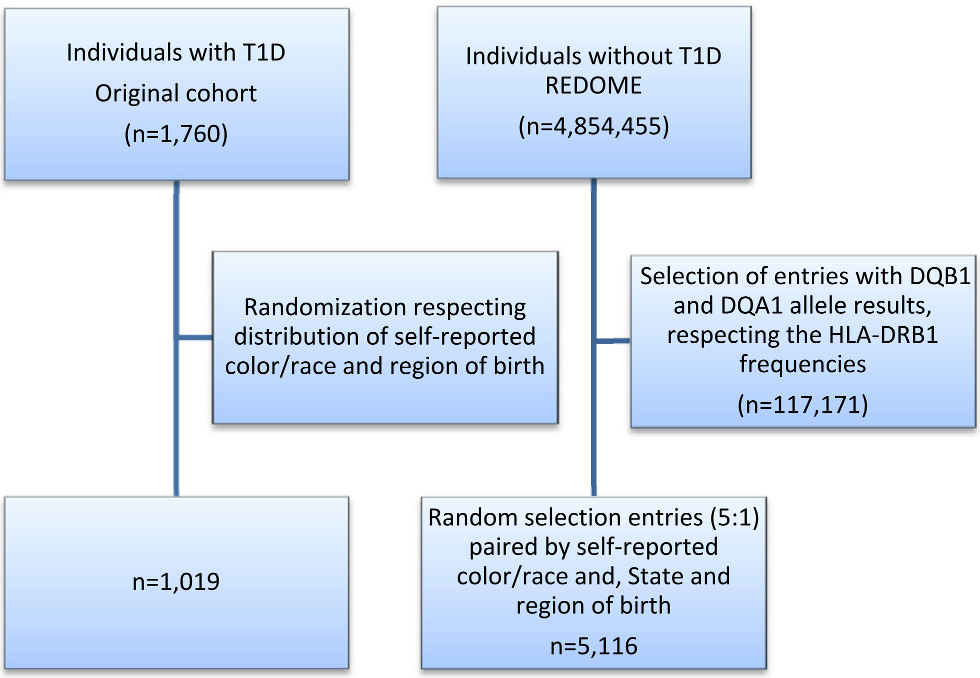

Table S1. Haplotypes in White self-reported color/race

DRB1~DQA1~DQB1	T1D (N)	T1D %	CONTROLS (N)	CONTROLS %	OR	CI 95%	p value
01:01~01:01g~05:01	42	4.04%	177	3.37%	1.21	0.86-1.70	.284
01:02~01:01g~05:01	27	2.60%	310	5.90%	0.42	0.28-0.63	.000
01:03~01:01g~05:01	3	0.29%	35	0.67%	0.43	0.13-1.40	.150
03:01~05:01g~02:01	326	31.35 %	371	7.07%	6.00	5.07-7.10	.000
03:02~04:01~04:02	1	0.10%	121	2.30%	0.04	0.01-0.29	.000
04:01~03:01g~03:01g	4	0.38%	44	0.84%	0.46	0.16-1.27	.125
04:01~03:01g~03:02	61	5.87%	53	1.01%	6.11	4.20-8.88	.000
04:02~03:01g~03:02	75	7.21%	99	1.89%	4.04	2.97-5.50	.000
04:03~03:01g~03:02	3	0.29%	15	0.29%	1.01	0.29-3.49	.988
04:04~03:01g~03:02	40	3.85%	60	1.14%	3.46	2.31-5.19	.000
04:05~03:01g~02:02	7	0.67%	8	0.15%	4.44	1.61-12.27	.002
04:05~03:01g~03:02	97	9.33%	112	2.13%	4.72	3.56-6.25	.000
04:06~03:01g~04:02	1	0.10%	19	0.36%	0.26	0.03-1.98	.164
04:07~03:01g~03:01g	2	0.19%	27	0.51%	0.37	0.09-1.57	.161
04:07~03:01g~03:02	5	0.48%	17	0.32%	1.49	0.55-4.04	.433
04:11~03:01g~03:02	6	0.58%	120	2.29%	0.25	0.11-0.56	.000
07:01~02:01~02:02	59	5.67%	586	11.16%	0.48	0.36-0.63	.000
07:01~02:01~03:03	0	0.00%	100	1.90%			
08:01~04:01~04:02	16	1.54%	92	1.75%	0.88	0.51-1.50	.628
08:02~04:01~04:02	3	0.29%	67	1.28%	0.22	0.07-0.71	.006
08:03~06:01~03:01g	0	0.00%	31	0.59%			
08:04~04:01~03:01g	4	0.38%	18	0.34%	1.12	0.38-3.32	.835
08:04~04:01~04:02	1	0.10%	30	0.57%	0.17	0.02-1.23	.046
08:07~04:01~04:02	1	0.10%	30	0.57%	0.17	0.02-1.23	.046
09:01~03:01g~02:02	12	1.15%	20	0.38%	3.05	1.49-6.26	.001
09:01~03:01g~03:03	4	0.38%	43	0.82%	0.47	0.17-1.30	.137
10:01~01:01g~05:01	10	0.96%	95	1.81%	0.53	0.27-1.01	.051
11:01~01:02~05:02	1	0.10%	27	0.51%	0.19	0.02-1.37	.064
11:01~01:02~06:02	2	0.19%	110	2.10%	0.09	0.02-0.36	.000
11:01~05:01g~03:01g	12	1.15%	204	3.89%	0.29	0.16-0.52	.000
11:02~05:01g~03:01g	7	0.67%	72	1.37%	0.49	0.22-1.06	.065
11:03~05:01g~03:01g	4	0.38%	37	0.70%	0.54	0.19-1.53	.241
11:04~05:01g~03:01g	9	0.87%	95	1.81%	0.47	0.24-0.94	.029
12:01g~01:01g~05:01	2	0.19%	19	0.36%	0.53	0.12-2.28	.386
12:01g~05:01g~03:01g	10	0.96%	26	0.50%	1.95	0.94-4.06	.069
12:02~06:01~03:01g	0	0.00%	20	0.38%			
13:01~01:03~06:03	24	2.31%	385	7.33%	0.3	0.20-0.45	.000
13:02~01:02~06:04	34	3.27%	123	2.34%	1.41	0.96-2.07	.080
13:02~01:02~06:09	7	0.67%	44	0.84%	0.8	0.36-1.78	.588
13:02~01:03~06:03	1	0.10%	22	0.42%	0.23	0.03-1.70	.115
13:03~05:01g~03:01g	6	0.58%	46	0.88%	0.66	0.28-1.54	.330
14:02~05:01g~03:01g	0	0.00%	123	2.34%			
14:06~05:01g~03:01g	0	0.00%	23	0.44%			

14:54~01:01g~05:03	0	0.00%	37	0.70%			
15:01~01:02~06:02	9	0.87%	255	4.86%	0.17	0.09-0.33	.000
15:02~01:03~06:01	1	0.10%	27	0.51%	0.19	0.02-1.37	.064
15:03~01:02~06:02	7	0.67%	175	3.33%	0.2	0.09-0.42	.000
16:01~01:02~05:02	10	0.96%	132	2.51%	0.38	0.20-0.72	.002
16:02~01:02~05:02	1	0.10%	32	0.61%	0.16	0.02-1.15	.036
16:02~05:01g~03:01g	0	0.00%	34	0.65%			
Others	83	7.98%	572	10.90%	0.71	0.56-0.90	.005

T1D = type 1 diabetes mellitus; n = number of individuals; OR = odds ratio; CI = confidence interval; Sixty-eight haplotypes with total number in patients plus controls greater than 18 were included (0.3%). P required for statistical significance after Bonferroni correction for multiple tests < 0.00074. Rare alleles were included in others.

Table S2. Haplotypes by Black self-reported color/race

DRB1~DQA1~DQB1	T1D (188)	T1D %	CONTROLS (928)	CONTROLS %	OR	CI 95%	p value
01:01~01:01g~05:01	9	4.79%	23	2.48%	1.98	0.90-4.35	.084
01:02~01:01g~05:01	3	1.60%	48	5.17%	0.3	0.09-0.96	.032
03:01~05:01g~02:01	45	23.94%	55	5.93%	4.99	3.24-7.69	.000
03:02~04:01~04:02	4	2.13%	46	4.96%	0.42	0.15-1.17	.087
04:01~03:01g~03:02	4	2.13%	6	0.65%	3.34	0.93-11.95	.049
04:02~03:01g~03:02	5	2.66%	17	1.83%	1.46	0.53-4.02	.457
04:05~03:01g~03:02	21	11.17%	18	1.94%	6.36	3.32-12.19	.000
04:11~03:01g~03:02	1	0.53%	28	3.02%	0.17	0.02-1.27	.051
07:01~02:01~02:02	13	6.91%	91	9.81%	0.68	0.37-1.25	.214
08:01~04:01~04:02	1	0.53%	18	1.94%	0.27	0.04-2.04	.174
09:01~03:01g~02:02	15	7.98%	12	1.29%	6.62	3.04-14.38	.000
10:01~01:01g~05:01	3	1.60%	16	1.72%	0.92	0.27-3.20	.901
11:01~01:02~06:02	2	1.06%	51	5.50%	0.18	0.04-0.77	.009
11:01~05:01g~03:01g	2	1.06%	22	2.37%	0.44	0.10-1.90	.260
13:01~01:03~06:03	3	1.60%	56	6.03%	0.25	0.08-0.81	.013
13:02~01:02~06:04	7	3.72%	21	2.26%	1.67	0.7-3.99	.243
13:02~01:02~06:09	1	0.53%	23	2.48%	0.21	0.03-1.57	.093
14:02~05:01g~03:01g	1	0.53%	19	2.05%	0.26	0.03-1.92	.153
15:01~01:02~06:02	1	0.53%	23	2.48%	0.21	0.03-1.57	.093
15:03~01:02~06:02	4	2.13%	85	9.16%	0.22	0.08-0.59	.001
Others	43	22.80%	250	26.93%	0.8	0.56-1.16	.248

T1D = type 1 diabetes mellitus; n = number of individuals; OR = odds ratio; CI = confidence interval; Sixty-eight haplotypes with total number in patients plus controls greater than 18 were included (0.3%). P required for statistical significance after Bonferroni correction for multiple tests < 0.00074. Rare alleles were included in others.

Table S3. Haplotypes by Brown self-reported color/race

DRB1~DQA1~DQB1	T1D (764)	T1D %	CONTROLS (3896)	CONTROLS %	OR	CI 95%	p value
**01:01~01:01g~05:01**	22	2.88%	105	2.70%	1.07	0.67-1.71	.775
**01:02~01:01g~05:01**	24	3.14%	214	5.49%	0.56	0.36-0.86	.007
**01:03~01:01g~05:01**	0	0.00%	26	0.67%			
**03:01~05:01g~02:01**	208	27.23%	235	6.03%	5.83	4.74-7.17	.000
**03:02~04:01~04:02**	4	0.52%	136	3.49%	0.15	0.05-0.39	.000
**04:01~03:01g~03:01g**	5	0.65%	20	0.51%	1.28	0.48-3.41	.625
**04:01~03:01g~03:02**	36	4.71%	22	0.56%	8.71	5.09-14.89	.000
**04:02~03:01g~03:02**	47	6.15%	81	2.08%	3.09	2.14-4.46	.000
**04:03~03:01g~03:02**	4	0.52%	16	0.41%	1.28	0.43-3.83	.663
**04:03~03:01g~03:04**	2	0.26%	1	0.03%	10.22	0.93-112.88	.019
**04:04~03:01g~03:02**	25	3.27%	35	0.90%	3.73	2.22-6.27	.000
**04:05~03:01g~03:02**	84	10.99%	75	1.93%	6.29	4.56-8.68	.000
**04:07~03:01g~03:01g**	1	0.13%	28	0.72%	0.18	0.02-1.33	.059
**04:07~03:01g~03:02**	3	0.39%	16	0.41%	0.96	0.28-3.29	.943
**04:11~03:01g~03:02**	6	0.79%	109	2.80%	0.27	0.12-0.63	.001
**07:01~02:01~02:02**	52	6.81%	421	10.81%	0.60	0.45-0.81	.001
**07:01~02:01~03:03**	4	0.52%	55	1.41%	0.37	0.13-1.02	.045
**08:01~04:01~04:02**	13	1.70%	59	1.51%	1.12	0.61-2.06	.701
**08:02~04:01~04:02**	2	0.26%	59	1.51%	0.17	0.04-0.70	.005
**08:03~06:01~03:01g**	1	0.13%	32	0.82%	0.16	0.02-1.16	.037
**08:04~04:01~03:01g**	3	0.39%	34	0.87%	0.45	0.14-1.46	.172
**08:04~04:01~04:02**	3	0.39%	44	1.13%	0.34	0.11-1.11	.062
**08:07~04:01~04:02**	2	0.26%	18	0.46%	0.57	0.13-2.44	.439
**09:01~03:01g~02:02**	27	3.53%	28	0.72%	5.06	2.97-8.64	.000
**09:01~03:01g~03:03**	4	0.52%	37	0.95%	0.55	0.19-1.54	.249
**10:01~01:01g~05:01**	10	1.31%	77	1.98%	0.66	0.34-1.28	.213
**11:01~01:02~05:02**	0	0.00%	20	0.51%			
**11:01~01:02~06:02**	3	0.39%	158	4.06%	0.09	0.03-0.29	.000
**11:01~05:01g~03:01g**	5	0.65%	95	2.44%	0.26	0.11-0.65	.002
**11:02~05:01g~03:01g**	3	0.39%	67	1.72%	0.22	0.07-0.72	.006
**11:04~05:01g~03:01g**	6	0.79%	19	0.49%	1.61	0.64-4.06	.303
**12:01g~01:01g~05:01**	2	0.26%	32	0.82%	0.32	0.08-1.32	.097
**13:01~01:02~05:01**	1	0.13%	21	0.54%	0.24	0.03-1.80	.132
**13:01~01:03~06:03**	14	1.83%	226	5.80%	0.30	0.18-0.52	.000
**13:01~01:03~06:04**	2	0.26%	1	0.03%	10.22	0.93-112.88	.019
**13:02~01:02~05:01**	5	0.65%	16	0.41%	1.60	0.58-4.37	.358
**13:02~01:02~06:04**	15	1.96%	98	2.52%	0.78	0.45-1.34	.364
**13:02~01:02~06:09**	7	0.92%	51	1.31%	0.70	0.31-1.54	.371
**13:03~05:01g~03:01g**	4	0.52%	29	0.74%	0.70	0.25-2.00	.506
**14:02~05:01g~03:01g**	0	0.00%	84	2.16%			
**14:06~05:01g~03:01g**	3	0.39%	18	0.46%	0.85	0.25-2.89	.794
**14:54~01:01g~05:03**	0	0.00%	36	0.92%			
**15:01~01:02~06:02**	6	0.79%	127	3.26%	0.23	0.10-0.53	.000
**15:03~01:02~06:02**	7	0.92%	237	6.08%	0.14	0.07-0.30	.000
**16:01~01:02~05:02**	7	0.92%	69	1.77%	0.51	0.23-1.12	.088

16:02~01:02~05:02	2	0.26%	30	0.77%	0.34	0.08-1.42	.120
16:02~05:01g~03:01g	4	0.52%	42	1.08%	0.48	0.17-1.35	.156
Others	76	9.94%	437	11.21%	0.87	0.68-1.13	.306

T1D = type 1 diabetes mellitus; n = number of individuals; OR = odds ratio; CI = confidence interval; Sixty-eight haplotypes with total number in patients plus controls greater than 18 were included (0.3%). P required for statistical significance after Bonferroni correction for multiple tests < 0.00074. Rare alleles were included in others.

Table S4. Haplotypes by Indigenous self-reported color/race

DRB1~DQA1~DQB1	T1D (N)	T1D (%)	Controls (N)	Controls (%)	OR	CI 95%	p value
01:01~01:01g~05:01	1	4.17%	5	8.06%	0.5	0.05-4.48	.524
01:02~01:01g~05:01	0	0.00%	2	3.23%			
03:01~05:01g~02:01	6	25.00%	3	4.84%	6.56	1.49-28.88	**.006**
03:02~04:01~04:02	1	4.17%	1	1.61%	2.65	0.16-44.18	.481
04:01~03:01g~03:01g	0	0.00%	1	1.61%			
04:02~03:01g~03:02	3	12.50%	1	1.61%	8.71	0.86-88.40	**.032**
04:04~03:01g~03:02	2	8.33%	2	3.23%	2.73	0.36-20.56	.313
04:04~03:01g~04:02	0	0.00%	2	3.23%			
04:05~03:01g~03:02	3	12.50%	2	3.23%	4.29	0.67-27.45	.099
07:01~02:01~02:02	0	0.00%	5	8.06%			
07:01~02:01~03:03	0	0.00%	2	3.23%			
07:01~03:01g~02:02	2	8.33%	0	0.00%			
07:15/07:21~02:01~02:02	0	0.00%	1	1.61%			
08:01~04:01~04:02	0	0.00%	2	3.23%			
08:04~04:01~04:02	0	0.00%	2	3.23%			
08:07~04:01~04:02	0	0.00%	2	3.23%			
09:01~03:01g~02:02	2	8.33%	0	0.00%			
11:01~01:02~05:02	0	0.00%	1	1.61%			
11:01~01:02~06:02	0	0.00%	3	4.84%			
11:01~05:01g~03:01g	0	0.00%	2	3.23%			
11:02~05:01g~03:01g	0	0.00%	1	1.61%			
11:02~05:10~03:01g	1	4.17%	0	0.00%			
13:01~01:02~05:01	1	4.17%	0	0.00%			
13:01~01:03~06:02	0	0.00%	1	1.61%			
13:01~01:03~06:03	1	4.17%	5	8.06%	0.5	0.05-4.48	.524
13:02~01:02~06:04	0	0.00%	4	6.45%			
13:03~05:01g~03:01g	0	0.00%	1	1.61%			
14:02~05:01g~03:01g	0	0.00%	1	1.61%			
14:02~05:01g~03:02	0	0.00%	1	1.61%			
15:01~01:02~05:03	0	0.00%	1	1.61%			
15:01~01:02~06:02	0	0.00%	4	6.45%			
15:03~01:02~06:02	0	0.00%	3	4.84%			
16:01~01:02~06:10	0	0.00%	1	1.61%			
16:02~03:01g~03:02	1	4.17%	0	0.00%			

T1D = type 1 diabetes mellitus; n = number of individuals; OR = odds ratio; CI = confidence interval

Table S5. Haplotypes by Yellow self-reported color/race

DRB1~DQA1~DQB1	T1D (N)	T1D (%)	Controls (N)	Controls (%)	OR	CI 95%	p value
01:01~01:01g~05:01	1	4.55%	5	5.21%	0.87	0.10-7.81	.898
01:02~01:01g~05:01	0	0.00%	5	5.21%			
01:03~01:01g~05:01	0	0.00%	1	1.04%			
01:03~01:03~06:03	1	4.55%	0	0.00%			
03:01~05:01g~02:01	5	22.73%	7	7.29%	3.74	1.06-12.18	**.031**
03:02~04:01~04:02	0	0.00%	2	2.08%			
04:01~03:01g~03:01g	0	0.00%	1	1.04%			
04:01~03:01g~03:02	1	4.55%	0	0.00%			
04:02~03:01g~03:02	0	0.00%	1	1.04%			
04:05~03:01g~03:02	0	0.00%	3	3.13%			
04:05~03:01g~04:01	1	4.55%	6	6.25%	0.71	0.08-6.25	.760
04:07~03:01g~03:01g	0	0.00%	1	1.04%			
04:07~03:01g~03:02	0	0.00%	2	2.08%			
04:08~03:01g~03:01g	0	0.00%	1	1.04%			
04:11~03:01g~03:02	1	4.55%	2	2.08%	2.24	0.19-25.85	.508
07:01~02:01~02:02	1	4.55%	10	10.42%	0.41	0.05-3.38	.393
07:01~02:01~03:02	1	4.55%	0	0.00%			
07:01~02:01~03:03	0	0.00%	1	1.04%			
07:01~03:01g~02:02	0	0.00%	1	1.04%			
08:02~04:01~04:01	0	0.00%	1	1.04%			
08:03~01:03~06:01	0	0.00%	2	2.08%			
09:01~03:01g~02:02	2	9.09%	0	0.00%			
09:01~03:01g~03:03	2	9.09%	1	1.04%	9.5	0.82-109.91	**.031**
11:01~01:02~05:02	0	0.00%	2	2.08%			
11:01~01:02~06:02	1	4.55%	4	4.17%	1.09	0.12-10.31	.937
11:01~05:01g~03:01g	0	0.00%	3	3.13%			
11:04~01:03~06:03	0	0.00%	1	1.04%			
11:04~05:01g~03:01g	0	0.00%	1	1.04%			
12:01g~03:01g~03:02	1	4.55%	0	0.00%			
13:01~01:03~06:03	1	4.55%	4	4.17%	1.09	0.12-10.31	.937
13:01~01:03~06:04	0	0.00%	1	1.04%			
13:01~03:01g~03:03	0	0.00%	1	1.04%			
13:02~01:02~05:01	0	0.00%	4	4.17%			
13:02~01:02~06:04	1	4.55%	2	2.08%	2.24	0.19-25.85	.508
13:02~01:02~06:09	0	0.00%	4	4.17%			
13:03~02:01~02:02	0	0.00%	1	1.04%			
13:03~03:01g~03:01g	1	4.55%	0	0.00%			
14:03~05:01g~03:01g	0	0.00%	1	1.04%			
14:04~01:01g~05:03	1	4.55%	0	0.00%			
15:01~01:02~06:02	0	0.00%	6	6.25%			
15:01~01:03~06:01	0	0.00%	1	1.04%			
15:02~01:03~06:01	0	0.00%	4	4.17%			
15:03~01:02~06:02	0	0.00%	3	3.13%			

T1D = type 1 diabetes mellitus; n = number of individuals; OR = odds ratio; CI = confidence interval

Table S6. Frequency distribution (n and %) of risk haplotypes associated with type 1 diabetes mellitus (T1D)# in all Brazilian regions

	Risk Haplotypes (*DRB1~DQA1~DQB1*)						
Regions	*03:01~ 05:01g ~02:01*	*04:01~ 03:01g ~03:02*	*04:02~ 03:01g ~03:02*	*04:04~ 03:01g ~03:02*	*04:05~ 03:01g ~02:02*	*04:05~ 03:01g ~03:02*	*09:01~ 03:01g ~02:02*
Center-West-Oeste (n=81)	35 (43.2%)	4 (4.94%)	9 (11.1%)	6 (7.41%)	3 (3.7%)	19 (23.4%)	5 (6.2%)
Northeseast (n=318)	149 (46.8%)	34 (10.6%)	36 (11.3%)	13 (4.09%)	2 (6.3%)	71 (22.3%)	25 (7.8%)
North (n=44)	21 (47.7%)	2 (4.55%)	5 (11.3%)	4 (9.09%)	3 (6.82%)	7 (15.9%)	1 (2.8%)
Southeast (n=429)	215 (50.1%)	40 (9.32%)	60 (13.9%)	30 (6.99%)	10 (2.3%)	75 (17.4%)	25 (5.8%)
South(n=147)	80 (54.4%)	20 (13.6%)	18 (12.2%)	13 (8.84%)	4(2.7%)	30 (20.4%)	2 (1.4%)
Q2 -df (p) T1D df=4	***3.64 (0.456)***	***6.33 (0.175)***	***1.46 (0.832)***	***5.15 (0.271)***	***9.24 (0.055)***	***3.86 (0.425)***	***8.93 (0.062)***

# - Odds ratio > 3.0; Q2 -Chi-squared, df – degree of freedom. T1D = type 1 diabetes
